# Chinese herb pair Paeoniae Radix Alba and Atractylodis Macrocephalae Rhizoma suppresses LPS-induced inflammatory response through inhibiting MAPK and NF-κB pathway

**DOI:** 10.1186/s13020-019-0224-2

**Published:** 2019-01-29

**Authors:** Yangyang Zhou, Hongxun Tao, Anqi Wang, Zhangfeng Zhong, Xu Wu, Mei Wang, Zhaoxiang Bian, Shengpeng Wang, Yitao Wang

**Affiliations:** 1 State Key Laboratory of Quality Research in Chinese Medicine, Institute of Chinese Medical Sciences, University of Macau, Avenida da Universidade, Taipa, Macao China; 2 Laboratory of Molecular Pharmacology, Department of Pharmacology, School of Pharmacy, Southwest Medical University, Luzhou, Sichuan China; 3 Leiden University European Center for Chinese Medicine and Natural Compounds, Institute of Biology, Leiden University, Leiden, The Netherlands; 4 School of Chinese Medicine, Hong Kong Baptist University, Kowloon, Hong Kong, China

**Keywords:** Radix Paeoniae Alba, Rhizoma Atractylodis Macrocephalae, Herb pair, Inflammatory

## Abstract

**Background:**

The combination of Radix Paeoniae Alba (RPA) and Rhizoma Atractylodis Macrocephalae (RAM) has long been used as a classic herb pair for the treatment of gynecologic and gastrointestinal diseases, but the underlying mechanisms of the herb pair remain unknown. This study aims to explore the anti-inflammatory potentials of RPA–RAM herb pair and to elucidate the underlying mechanisms.

**Methods:**

The bioactive parts of RPA–RAM were extracted and screened through the inhibitory effects against nitric oxide (NO) production. The effects of optimized RPA–RAM extracts (OPAE) on inflammation-associated mediators were investigated by Western blotting, real-time quantitative PCR (RT-qPCR), Enzyme-linked immunosorbent (ELISA) and immunofluorescence staining.

**Results:**

OPAE potently suppressed the productions of NO, TNF-α, IL-6 and MCP-1 in lipopolysaccharide (LPS)-induced RAW 264.7 macrophages, concentration-dependently inhibited protein level of inducible nitric oxide synthase (iNOS), dramatically downregulated mRNA expression of iNOS, TNF-α, IL-6 and MCP-1. In addition, OPAE significantly prevented phosphorylation and degradation of inhibitory kappa Bα (IκBα) and subsequently restrained the nuclear translocation of NF-κB p65. Pretreatment with OPAE also attenuated the LPS-induced phosphorylation of ERK, JNK and p38.

**Conclusions:**

Our findings demonstrated that OPAE suppressed inflammatory responses in LPS-stimulated RAW 264.7 macrophages by decreasing critical molecules involved in MAPK and NF-κB pathway, suggesting that the herb pair could be a promising therapeutic candidate for inflammation-related diseases.

**Electronic supplementary material:**

The online version of this article (10.1186/s13020-019-0224-2) contains supplementary material, which is available to authorized users.

## Background

Inflammation, generally classified as acute and chronic, is a protective process of the host to injuries or stimuli, but dysregulated responses also cause disorders and diseases including arthritis, autoimmune diseases, cancer, diabetes, neurological diseases [1]. Macrophages play a crucial role in scavenging pathogens, presenting antigens and secreting mediators in the initiation of immune responses and the development of inflammation [2, 3]. Lipopolysaccharide (LPS) is one of the predominant microbial initiators to active macrophages [4, 5], involving in recognition and binding to toll-like receptors (TLRs) in cellular membrane [6]; subsequent triggering the formation of active signaling proteins including mitogen-activating protein kinases (MAPKs; p38; extracellular signal-regulated kinases, ERK; c-Jun N-terminal kinase; JNK) [7], the IκB-kinase (IKK) complex, and IκBα [8]; translocation of transcriptional mediators (nuclear factor kappa B, NF-κB) into nucleus [9]; downstream excessive pro-inflammatory factors (nitric oxide, NO; tissue necrosis factor-α, TNF-α; interleukin-6, IL-6) [10, 11], and inducible enzymes (COX-2 and iNOS) [12, 13].

Herb pairs (combination of two herbs) are the most basic composition units of Chinese compound formulae, which remain therapeutic features and clinical significance in Chinese herbal medicine (CHM) [14]. Radix Paeoniae Alba (RPA, *Baishao* in Chinese) is the dried root of *Paeonia lactiflora* Pall. and Rhizoma Atractylodis Macrocephalae (RAM, *Baizhu* in Chinese) is the dried rhizome of *Atractylodes macrocephala* Koidz [15]. In the clinical practices of CHM, the herb pair consisting of RPA and RPM is well-known in numerous classic formulae such as *Baizhu Shaoyao San* (*Tongxie Yaofang,* TXYF), *Xiaoyao San* (SYS), *Renshen Yangrong Wan*, and *Danggui Shaoyao San* (DSS) for the synergistic effects on promoting “Qi” circulation, replenishing blood, nourishing liver, emolliating spleen, reliving pain and stopping diarrhea. Among them, TXYF have attracted wide interest in the treatment of irritable bowel syndrome (IBS) and inflammatory bowel diseases (IBD) [16, 17], DSS have been reported to exert anti-inflammatory effects for Alzheimer’s diseases (AD) therapy [18]. XYS regulated neuro-inflammatory responses in brain disorders [19]. However, the effects and mechanisms of paired PRA–PAM herb in inflammation regulation still remain unknown.

In order to understand the mechanisms of this herb pair, we firstly screened the PRA–PAM extracts (PAEs) isolated in different solvent systems through chemical and biological assessments. We next investigated the anti-inflammatory effects of OPAE using LPS-stimulated murine macrophage RAW264.7 cells. In addition, the underlying molecular mechanisms were further investigated in this study.

## Materials and methods

The Minimum Standards of Reporting Checklist contains details of the experimental design, and statistics, and resources used in this study (Additional file 1).

### Chemicals and reagents

Paeoniflorin, atractylenolide-I, atractylenolide-II and atractylenolide-III (HPLC ≥ 98%) was obtained from Must Bio-Technology Co., Ltd. (Chengdu, China). Lipopolysaccharide (LPS) from *Escherichia coli* (0111: B4), 3-(4,5-dimethylthiazol-2-yl)-2,5-diphenylte-trazolium bromide (MTT), Griess reagent, dimethyl sulfoxide (DMSO) were purchased from Sigma-Aldrich (CA, USA). Dulbecco’s modified Eagle’s medium (DMEM), fetal bovine serum (FBS), antibiotic–antimycotic solution and phosphate-buffered saline (PBS) were procured by Gibco (Thermo Fisher Scientific, USA). Sodium nitrite solution and RIPA lysis buffer was got from Beyotime Biotechnology (Shanghai, China). Mouse TNF-α, IL-6 and MCP-1 ELISA kits were ordered from Biolegend (CA, USA). Primary antibodies against iNOS, COX-2, NF-κB/p65, phospho-p65, IκBα, phospho-IκBα, p38, phospho-p38, ERK, phospho-ERK, JNK, phospho-JNK, GADPH (Glyceraldehyde 3-phosphate dehydrogenase), and peroxidase-conjugated secondary antibody were acquired from Cell Signaling Technology (CA, USA). All other chemicals were analytical-reagent grade.

### Cell culture

The Murine macrophage RAW 264.7 cell line was cultured in Dulbecco’s modified Eagle’s Medium (DMEM) supplemented 10% FBS, 100 EU mL^−1^ penicillin and 100 mg mL^−1^ streptomycin at 37 °C in a humidified environment containing 5% CO_2_.

### Preparation of PAEs

Crude herbal materials of the root barks of *Paeonia lactiflora* Pall. (Baishao in Chinese) and the rhizome of *Atractylodes macrocephala* Koidz. (Baizhu in Chinese) were purchased from Chinese herbal medicine market in Bozhou (Anhui, China) and Ya’an (Sichuan, China), respectively. After pulverized into powder and deposited at room temperature, 5 g of PRA and 5 g of AMR were extracted with 100 mL of different aqueous ethanol for 2 h using heating reflux at 100 °C. The extract solution was filtered and concentrated using vacuum evaporation; the residual solution was then lyophilized, yielding 3.70 g of dried powder (yield ratio 36.85%) in 25% ethanol–water extracted solution.

### Ultra-performance liquid chromatography (UPLC) of PAEs

Waters ACQUITY UPLC system (Waters Corp., MA, USA) with a PDA eλ detector was used for qualitative analysis of OPAE. Specimens were speared by a ACQUITY UPLC BEH C18 Column (2.1 × 50 mm, 1.7 µm, Waters Corp., USA). The mobile phase consisted of linear gradients of 0.2% (v/v) phosphoric acid (A) and acetonitrile (B): 0–25 min, 5–95% B (v/v); 25–30 min, 5% B (v/v). The flow rate was 0.3 mL/min, the injection volume was 10 μL, and the column was set at 35 °C. The data was collected and chromatogram was processed with MassLynx V4.1 software (Waters Corp., USA).

### Cell viability assay

MTT assay was conducted to examine cell viability. RAW264.7 cells (10^4^ cells per well) were seeded into 96-well plate overnight and then were treated with various concentrations of PAEs with or without LPS (1 μg mL^−1^) for 24 h. MTT solution was added at a final concentration of 500 μg mL^−1^ for another 4 h incubation at 37 °C. The supernatant was discarded and 100 μL DMSO was added to each well for formazan dissolution. The absorbance at 570 nm was read on a microplate reader (Molecular Devices, Sunnyvale, CA, USA). Cell viability was expressed as percentages relative to the untreated control.

### NO production determination

The amount of NO release was measured as concentration using the Griess reagent. RAW264.7 cells were seeded into 24-well plates (2 × 10^5^ cells per well) overnight and then treated with various concentrations of PAEs with LPS (1 μg mL^−1^) for 24 h. Then 50 μL of the supernatant and the equal volume of Griess reagent were mixed and reacted for 15 min, the absorbance was detected at 540 nm using a microplate reader. The inhibitory rates were calculated by regression analysis from a standard curve of sodium nitrite, by comparing with the LPS stimulated control group. The half maximal inhibitory concentration (IC_50_) values was calculated by the software GraphPad Prism 7.0. Briefly, individual concentration–response curves were generated by plotting the logarithm of tested drug concentrations (*X*) vs. corresponding inhibition percentages (*Y*) using least squares (ordinary) fit. Best fit IC_50_ values were calculated using log(inhibitor) vs. response-variable slope equation, where ${\text{Y}} = {\text{Bottom}} + ({\text{Top}} - {\text{Bottom}})/(1 + 10{\wedge} ({\text{LogIC}}_{50} - {\text{X}}) \times {\text{HillSlope}}))$.

### Measurement of cytokines

Pro-inflammatory cytokines accumulation were quantitated by enzyme-linked immunosorbent assay (ELISA) kits according to the manufacturer’s protocols. Briefly, cells (2 × 10^5^ cells per well) were co-treated with OPAE at different concentrations (0, 125, 250 and 500 μg mL^−1^) and LPS (1 μg mL^−1^) for 24 h in 24-well plates. The media were collected and centrifuged at 4000*g* for 5 min at 4 °C and the supernatant was appropriately diluted for further reaction.

### Western blot analysis

RAW 264.7 cells (6 × 10^6^ cells per dish) were seeded in 10 mm dishes overnight followed by pre-treatment with various concentration (0, 125, 250 and 500 μg mL^−1^) of OPAE for 4 h and then stimulation with LPS (1 µg mL^−1^) for another 30 min. For whole cell lysate preparation, cells were washed twice with ice-cold PBS and lysed on ice for 40 min using a lysis buffer including RIPA buffer (consists of 50 mM Tris pH 7.4, 150 mM NaCl, 1% NP-40, 0.1% SDS, 0.5% sodium deoxycholate) supplemented with Halt™ Protease Inhibitor Cocktail and Halt™ phosphatase inhibitor cocktail (Thermo Fisher Scientific, USA). Nuclear and cytoplasmic extractions were prepared using the NE-PER™ Nuclear and Cytoplasmic Extraction Reagents kit (Thermo Fisher Scientific, USA) according to the manufacturer’s instructions. Protein concentration was quantitated with a BCA protein assay kit (Thermo Fisher Scientific, USA). Equal amounts of protein (15–30 μg) were separated on sodium dodecyl sulfate polyacrylamide gel electrophoresis (SDS-PAGE), and transferred onto polyvinylidene difluoride (PVDF) membranes (Bio-Rad, CA, USA). After blocking with 5% nonfat milk for 2 h at room temperature, the membranes were subsequently incubated with specific primary antibodies (1:1000) overnight at 4 °C, followed by washed triplicately in TBST and incubated with appropriate horseradish peroxidase (HRP)-conjugated secondary antibodies (1:5000) for 2 h at room temperature. The proteins of interest were visualized using SuperSignal West Femto Maximum Sensitivity Substrate kit (Thermo Fisher Scientific, USA) and photographed by a ChemiDoc ™ MP Imaging System (Bio-Rad, CA, USA). Brand intensities were analyzed using Image J software and expressed as relative fold to reference proteins, the phosphorylated protein was then normalised to total proteins to show the fold increase in phosphorylation.

### Immunofluorescence staining

After incubation on chamber slides overnight, RAW 264.7 cells were pre-treated with OPAE at 500 μg mL^−1^ for 4 h and stimulated with LPS (1 µg mL^−1^) for another 30 min. Then cells were fixed with 4% formaldehyde for 15 min at room temperature, rinsed three times in PBS for 5 min each and blocked in blocking buffer (1 × PBS/1% BSA/0.1% Triton X-100) for 60 min. The slides were incubated with diluted primary antibody against NF-κB p65 (1:400) overnight at 4 °C, followed by washed with PBS and stained with Alexa Fluor 568 dye conjugated secondary antibody (1:500) with Hoechest 33342 dye (1:1000) in dark overnight at 4 °C. The Hoechst and Dylight fluorophores determined by confocal microscopy (Leica SP8, USA) detected changes in nuclear morphology (blue fluorescence) and NF-κB distribution (red fluorescence), respectively.

### RNA extraction and real-time qPCR

RAW 264.7 cells (2 × 10^5^ cells per well) were seeded into 24-well plates and co-treated with OPAE at high concentration (500 μg mL^−1^) with or without LPS (1 μg mL^−1^) for 24 h. Cells were collected and total RNA was isolated using RNAiso Plus regent (Takara Bio Inc., USA) according to the manufacturer’s instructions. The quantity of total RNA was detected at 260 and 280 nm. For each specimen, the cDNA was generated from 1 μg of total RNA using PrimeScript™ II 1st Strand cDNA Synthesis Kit (Takara Bio Inc., USA). The cDNA was conducted as a template with SYBR^®^ Premix Ex Taq™ (Takara Bio Inc., USA) and gene-specific primers. As previously described [20], the primer sequences were as follows: iNOS, 5′-CCAAGCCCTCACCTACTTCC-3′ (forward), and 5′-CTCTGAGGGCTGACACAA-GG-3′ (reverse); TNF-α, 5′-GAGAAAGTCAACCTCCTCTCTG-3′ (forward), and 5′-GAAGACTCCTCCCAGGTATATG-3′ (reverse); IL-6, 5′-CCAGAGATACAAA-GAAATGATGG-3′ (forward), and 5′-ACTCCAGAAGACCAGAGGAAAT-3′ (reverse); MCP-1, 5′-CAACTCTCACTGAAGCCAGCTC-3′ (forward), and 5′-TAGCTCTCCAGCCTACTCATTGG-3′ (reverse); GAPDH, 5′-TCACCACCATGG-AGAAGGC-3′ (forward), 5′-GCTAAGCAGTTGGTGGTGCA-3′ (reverse); NFκB, 5′-CATGCTGATGGAGTACCCTGAAGC-3′ (forward), 5′-CATGTCCGCAATGGAGGAGAAG-3′ (reverse); NFκBIA, 5′-TGGCAATCATCCACGAAGAGAAGC-3′ (forward), 5′-GCTCACAGGCAAGATGTAGAGGG-3′ (reverse). The real-time quantitative polymerase chain reaction (RT-qPCR) detections were performed on Mx3005P QPCR system (Agilent Technologies Inc., USA) under the following thermal cycler conditions: 30 s at 95 °C, 40 cycles of 5 s at 95 °C and 34 s at 60 °C, followed by a standard melting curve analysis used to verify the amplification of the single product per primer pair at the end of each experiment. The threshold cycle (C_T_) was calculated using MxPro-Mx3005P v4.10 Software (Agilent Technologies Inc., USA). GAPDH was used as the internal control for normalization, and 2^−ΔΔCT^ method was applied for increase or decrease fold.

### Statistical analysis

The experimental data were obtained from 3 to 5 independent and parallel tests and shown as mean ± standard deviation (SD). Statistical analysis was performed by one-way analysis of variance (ANOVA) using PRISMA software (GraphPad Software, Inc., San Diego, CA, version 7.0a) with Dunnet’s multiple comparisons test; *p* < 0.05 was considered as a statistically significant difference.

## Results

### Bioactivity-guided screening of PAEs with inhibition of NO production

**Fig. 1:**
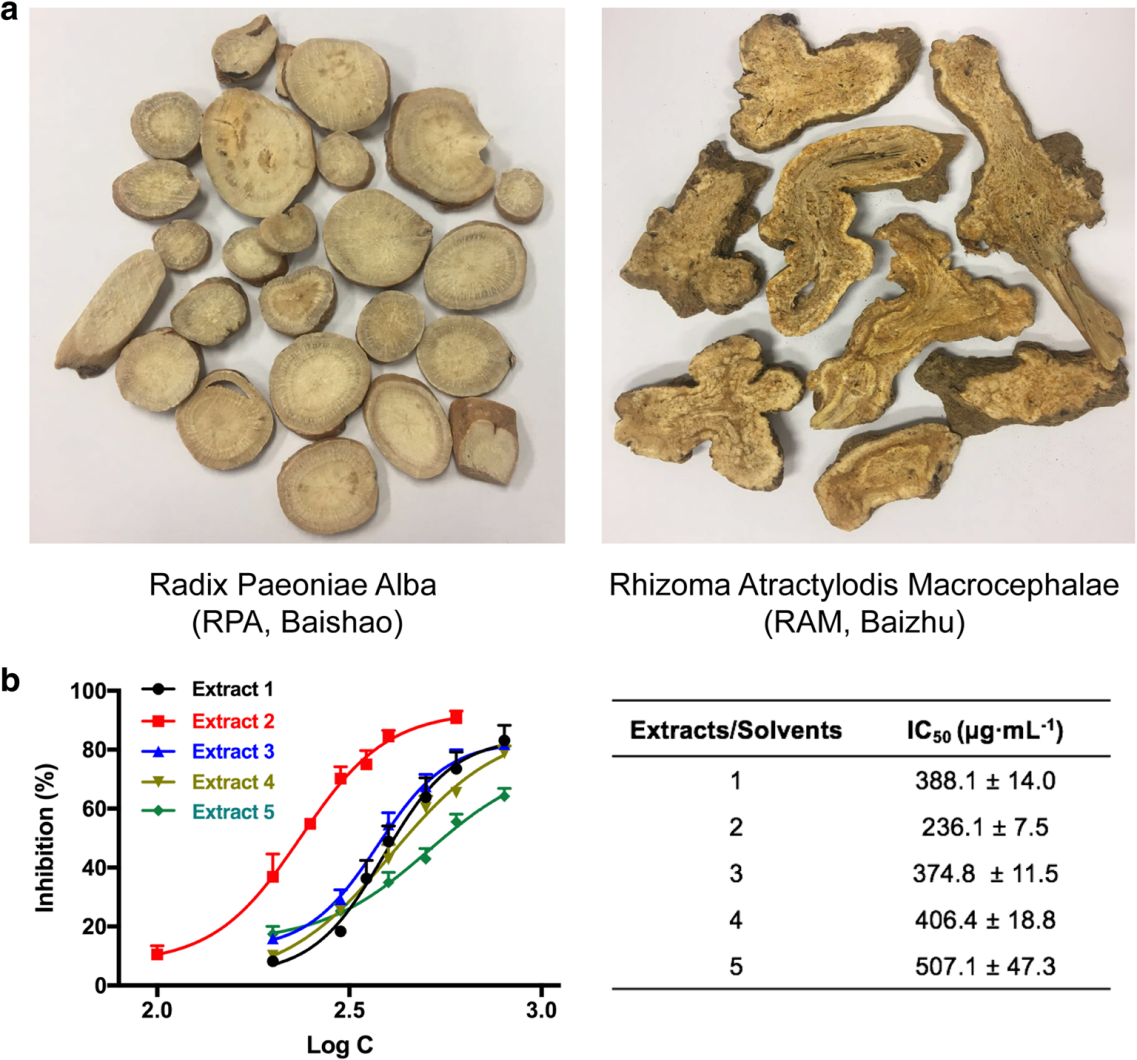
Bioactivity-guided extraction of RPA–RAM herb pair with inhibitory effect against NO production in LPS-stimulated RAW 264.7 macrophages. Cells were co-treated with PAEs at different concentrations ranging from 100 to 800 μg mL^−1^ and LPS (1 µg mL^−1^) for 24 h. **a** Picture of herb pair Radix Paeoniae Alba (RPA, Baishao) and Rhizoma Atractylodis Macrocephalae (RAM, Baizhu). **b** Concentration–response curves and IC_50_ values of NO inhibition for PAEs. Data are expressed as mean ± S.D., n = 3

Five gradient ratio of ethanol–water solvent systems (1–5, 100% H_2_O, 25% ethanol/H_2_O, 50% ethanol/H_2_O, 75% ethanol/H_2_O, and 95% ethanol/H_2_O) were set to obtain extracts of the RAM-RPA herb pair since the contrastive solubility of bioactive compounds. The IC_50_ values for NO production inhibition of the extracts were shown in Fig. 1b. The extracts isolated by 25% ethanol aqueous system showed the lowest IC_50_ value of 236.1 μg mL^−1^. We hence selected it as a major active fraction of RPA–RAM herb pair in following study.

### UPLC analysis for OPAE

**Fig. 2:**
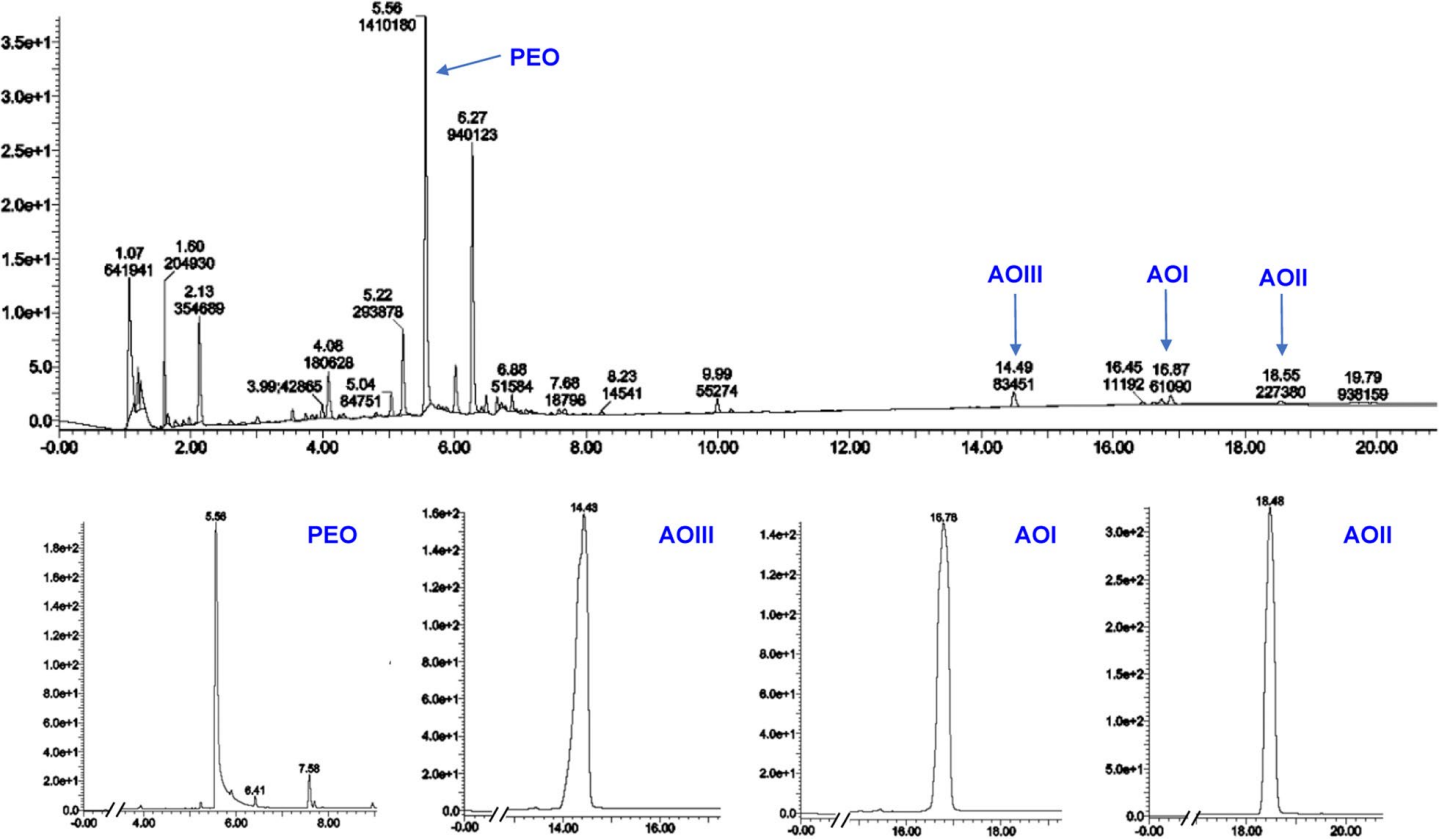
Analysis of OPAE through ultra-performance liquid chromatography (UPLC) equipped with a PDA eλ detector. Results are shown with matching standards of paeoniflorin (PEO), atractylenolide I (AOI), atractylenolide II (AOII) and atractylenolide III (AOIII). The UV absorption was monitored ranging from 200 to 400 nm

UPLC-UV analysis of OPAE showed a wide range of glycosides and lactones. Representative chromatogram in Fig. 2 confirmed the presences of active ingredients from PRA-RAM herbal pair, including paeoniflorin (PEO, retention time: 5.56 min), atractylenolide I (AOI, retention time: 16.87 min), atractylenolide II (AOII, retention time: 18.55 min) and atractylenolide III (AOIII, retention time: 14.49 min).

### Effects of OPAE on cell viability of RAW 264.7 murine macrophage cells

**Fig. 3:**
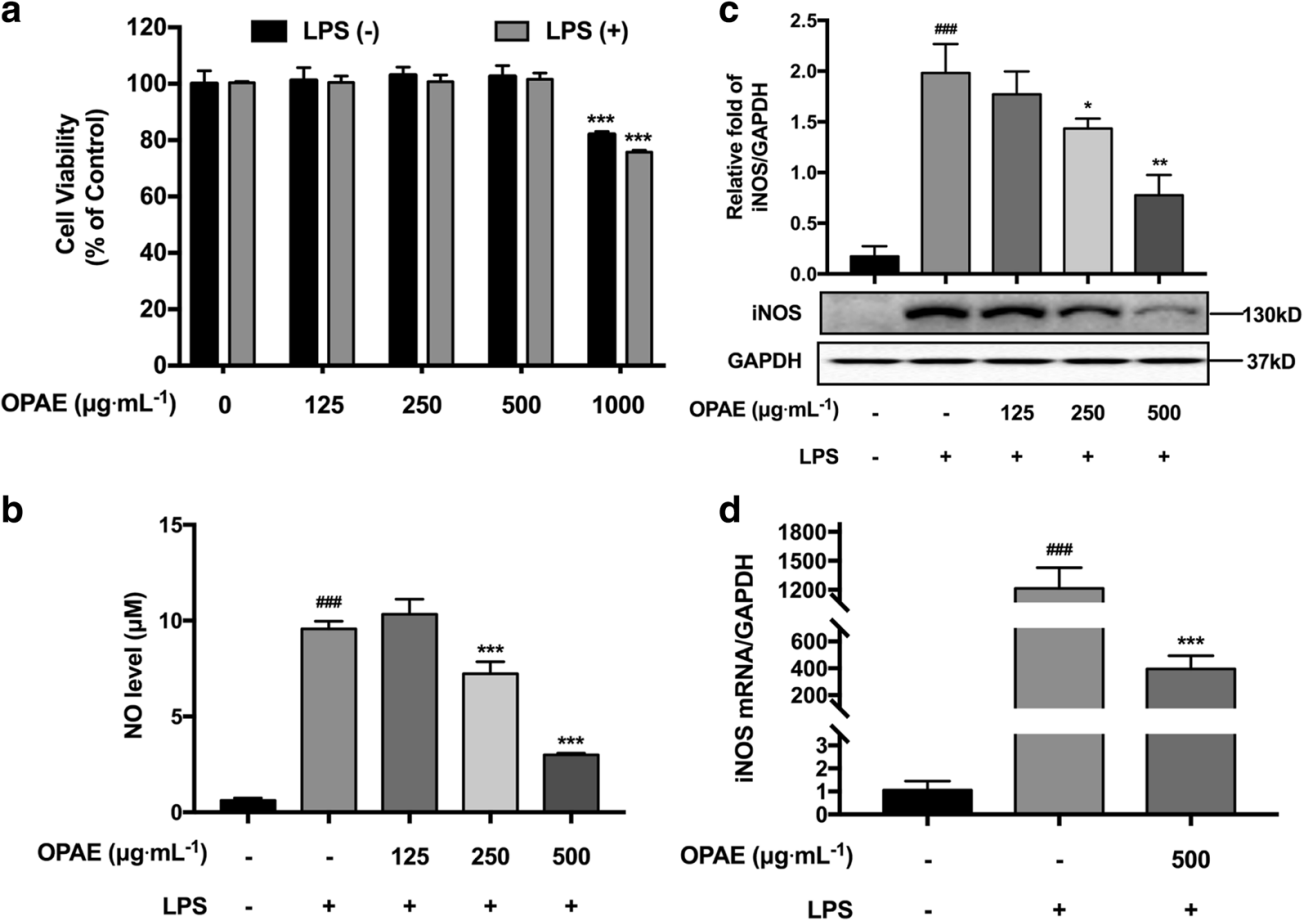
OPAE suppressed inflammatory response in LPS-stimulated RAW 264.7 macrophages. Cells were co-treated with OPAE at different concentrations (125, 250 and 500 mg mL^−1^) in the presence or absence LPS (1 µg mL^−1^) for 24 h. **a** Cell viability was measured with MTT assay. **b** NO production was determined by Griess reagent. **c** The expression of iNOS protein was determined by western blotting, GADPH was used as internal loading control. **d** The mRNA level of iNOS were analyzed by RT-qPCR, normalized to the GAPDH (n = 5). Data are expressed as mean ± S.D., n = 3. ^###^*p *<0.001 vs. Ctrl. group; *p <0.05, **p <0.01, ***p <0.001 vs. LPS group

When treated with OPAE at different concentration of 0, 125, 250, 500 and 1000 μg mL^−1^ in the presence of 1 μg mL^−1^ LPS, the RAW 264.7 cell viabilities were 100.36 ± 0.48%, 100.45 ± 2.31%, 100.74 ± 2.36%, 101.59 ± 2.24%, and 75.74% ± 0.67%, respectively. The result showed treatment with OPAE (125, 250 and 500 μg mL^−1^) and LPS (1 μg mL^−1^) had no obvious cytotoxicity on cell growth compared to control group (0 μg mL^−1^) (*p *> 0.05), significant difference was showed while exposure to 1000 μg mL^−1^ of OPAE (*p *< 0.001, Fig. 3a). Herein, cells were incubated with OPAE within the concentration of 500 μg mL^−1^ in the following experiments.

### OPAE prevented LPS-stimulated NO production and iNOS expression

As shown in Fig. 3b, NO release was remarkably increased in the presence of LPS (9.57 ± 0.20 μM, *p *< 0.001) compared with untreated cells (0.62 ± 0.06 μM). No obvious decrease of NO amount was obtained at concentration of 125 μg mL^−1^ (10.33 ± 0.40 μM, *p *> 0.05). When treated with 250 μg mL^−1^ (7.23 ± 0.31, *p *< 0.001) and 500 μg mL^−1^ (2.99 ± 0.05, *p *< 0.001), OPAE significantly decreased LPS-induced NO production in a concentration-dependent manner (*p *< 0.001). To determine whether the suppression of NO production is regulated by iNOS, the protein and mRNA expression were detected through western blot and RT-qPCR, respectively. Both protein (1.98 ± 0.17, *p *< 0.001, Fig. 3c) and mRNA expression (1212.00 ± 95.56, *p *< 0.001, Fig. 3d) of iNOS were dramatically up-regulated with LPS treatment. OPAE treatment significantly suppressed LPS-stimulated elevation of iNOS at mRNA level with high concentration (395.20 ± 40.18, *p *< 0.001). Consistently, OPAE suppressed the LPS-induced iNOS protein in a concentration-dependent manner (*p *< 0.01). The inhibitory effect was obtained at the concentration of 250 μg mL^−1^ (27.61%, *p *< 0.05) and 500 μg mL^−1^ (61.85%, *p *< 0.01), respectively.

### OPAE attenuated LPS-activated pro-inflammatory cytokines

**Fig. 4:**
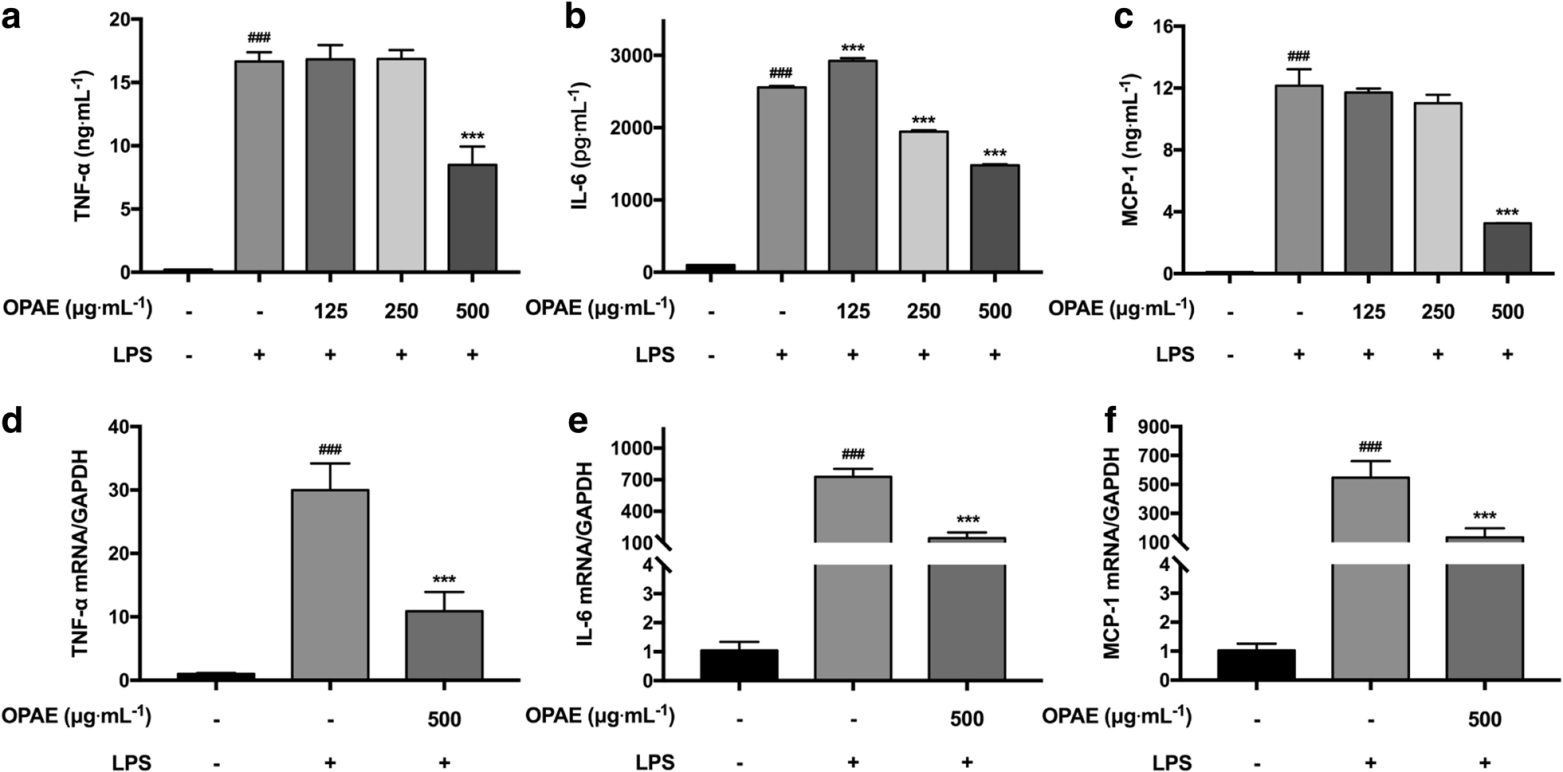
OPAE suppressed inflammatory cytokines release in LPS-stimulated RAW 264.7 macrophages. Cells were co-treated with OPAE at different concentrations (125, 250 and 500 mg mL^−1^) in the presence or absence of LPS (1 µg mL^−1^) for 24 h. The levels of **a** TNF-α, **b** IL-6 and **c** MCP-1 were determined by ELISA kit (n = 3). The mRNA level of **d** TNF-α, **e** IL-6 and **f** MCP-1 were analyzed by RT-qPCR, normalized to the GAPDH (n = 5). Data are expressed as mean ± S.D. ^###^*p *<0.001 vs. Ctrl. group, ***p <0.001 vs. LPS group

TNF-α, IL-6 and MCP-1 are representative pro-inflammatory cytokines secreted by macrophages, and all these cytokines were strongly increased following the treatment with LPS. The production of these cytokines was investigated using ELISA assay. OPAE visibly reduced the secretion of IL-6 in a concentration-dependent manner (Fig. 4b). The inhibition rate with co-treatment of OPAE at 500 μg mL^−1^ was 48.96% for TNF-α (Fig. 4a), 42.05% for IL-6 and 73.21% for MCP-1 (Fig. 4c), respectively. Consistent with the results of cytokines release, the relative mRNA expression quantitated by RT-qPCR revealed the inhibitory effect of OPAE in LPS-activated RAW 264.7 cells. The percent inhibition at high concentration of OPAE (500 μg mL^−1^) were 63.70% for TNF-α (Fig. 4e), 79.89% for IL-6 (Fig. 4f) and 75.48% for MCP-1 (Fig. 4g), respectively.

### OPAE reduced activation of NF-κB signaling in LPS-induced RAW 264.7 cells

**Fig. 5:**
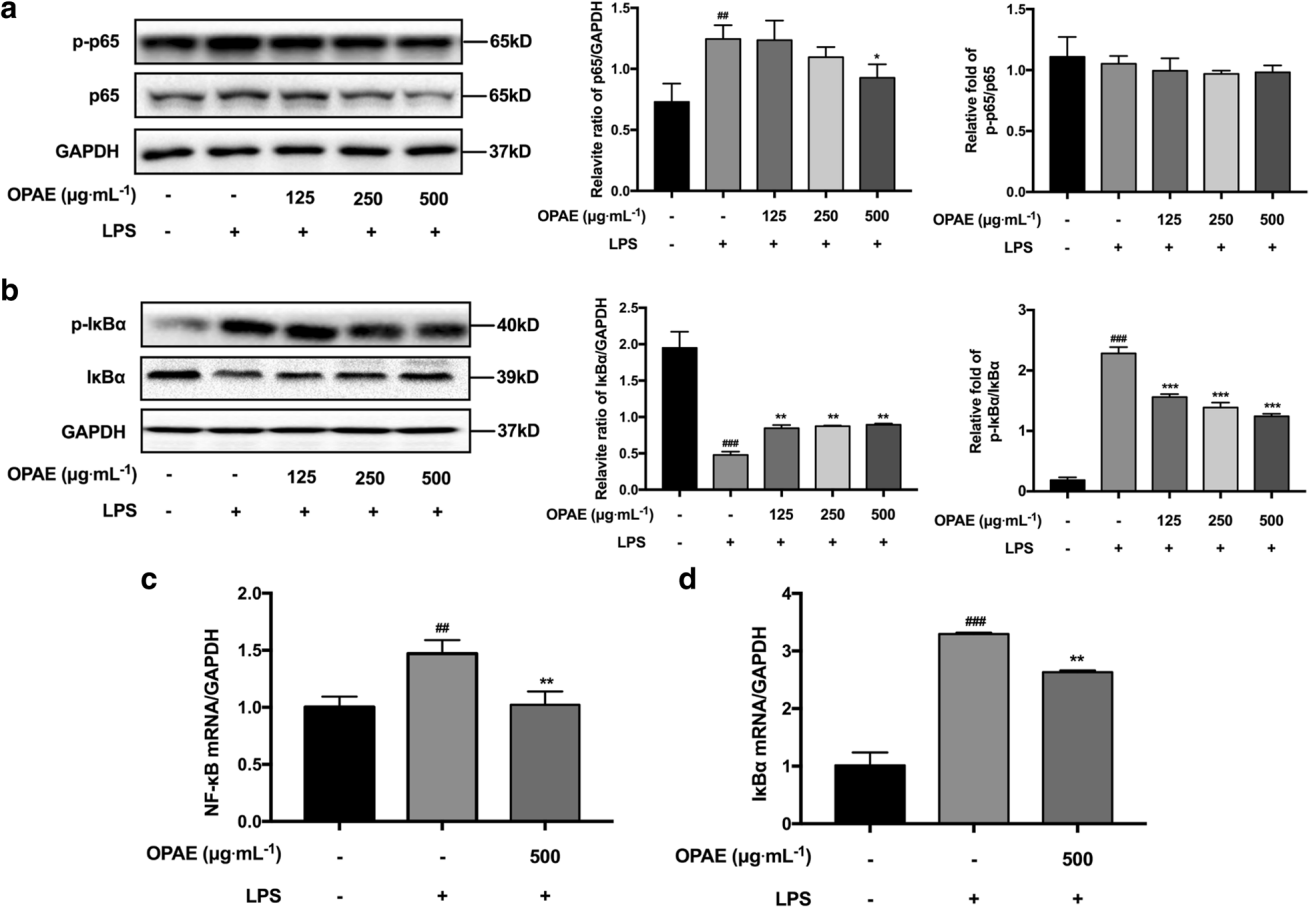
OPAE reduced NF-κB activation in LPS-treated RAW264.7 macrophages. Cells were pre-treated with OPAE at different concentrations (125, 250, and 500 mg mL^−1^) for 4 h and then stimulated with LPS (1 µg mL^−1^) for another 30 min. The protein expression of **a** p65 and p-p65, **b** IκBα and p-IκBα, was determined by western blotting, GAPDH was used as internal loading control. The mRNA expression of **c** NF-κB and **d** NFκBIA was analyzed by RT-qPCR, normalized to the GAPDH. Data are expressed as mean ± S.D., n = 3. ^##^*p *<0.01, ^###^*p *<0.001 vs. Ctrl. group; *p <0.05, **p <0.01, ***p <0.001 vs. LPS group

**Fig. 6:**
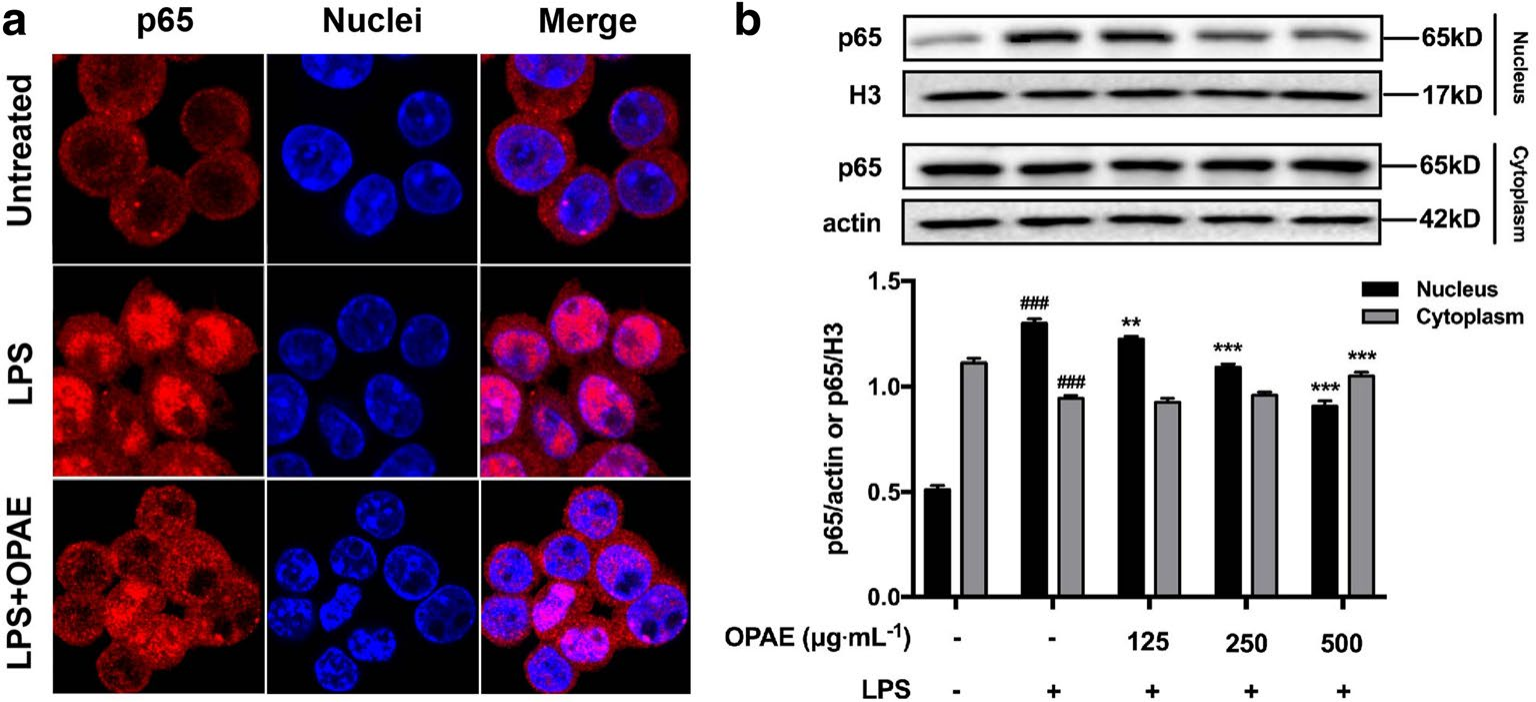
OPAE reduced NF-κB translocation into nucleus in LPS-treated RAW264.7 macrophages. Cells were pre-treated with OPAE at different concentrations (125, 250, and 500 mg mL^−1^) for 4 h and then stimulated with LPS (1 µg mL^−1^) for another 30 min. **a** The transcription factor, p65, was stained with primary p65 antibody followed by Alexa Fluor 568 dye conjugated secondary antibody (red fluorescence) and Hoechst 33342 dye (blue fluorescence), sequentially. **b** Nuclear and cytosolic proteins were subjected to Western blot analysis with the indicated anti-bodies. β-actin and histon H3 were used as internal controls for the cytosolic and nuclear fractions, respectively. Data are expressed as mean ± S.D. ^###^*p *<0.001 vs. Ctrl. group, **p <0.01, ***p <0.001 vs. LPS group

Regarding to the NF-κB pathway, we showed the remarkably inhibitory effects of OPAE on the phosphorylation of IκBα in a concentration-dependent manner (Fig. 5b), the relative fold increase of p-IκBα/IκBα was 1.56 ± 0.05 (125 μg mL^−1^, *p *< 0.001), 1.39 ± 0.08 (250 μg mL^−1^, *p *< 0.001) and 1.24 ± 0.24 (500 μg mL^−1^, *p *< 0.001), while that of LPS-alone treated group was 2.28 ± 0.10. As shown in Fig. 5b, LPS treatment strongly induced the degradation of IκBα, and OPAE treatment rescued it with a slight but significant effect (*p *< 0.05). Although OPAE observably reduced p65 level at the concentration of 250 μg mL^−1^ (*p *< 0.05, Fig. 5a), there was no obvious decrease of the phosphorylated p65 (Fig. 5a). The relative mRNA expression of NF-κB (Fig. 5c) and IκBα (Fig. 5d) was increased when exposure to LPS (1 μg mL^−1^) for 30 min, and pre-treatment with OPAE at 500 μg mL^−1^ for 4 h could significantly suppressed the LPS-stimulated increase at transcription level (*p *< 0.01). We subsequently investigated whether OPAE affected the translocation of p65 into nucleus, which is a critical step in the activation of NF-κB pathway. The immunostaining result shows the retention of p65 (red fluorescence) in the cytoplasm in untreated cells, whereas the nuclear distribution in LPS-induced cells, and OPAE treatment at 500 μg mL^−1^ significantly reduced the nuclear accumulation of p65 (Fig. 6a). As shown by western blot result (Fig. 6b), LPS stimulation led to an increase of p65 in the nucleus but a decrease in the cytoplasm (*p *< 0.001), consistent with immunofluorescence, pretreatment with OPAE (500 μg mL^−1^, *p *< 0.001) significantly reduced the translocation of p65 into nucleus.

### OPAE suppressed MAPKs pathway in LPS-challenged RAW 264.7 cells

**Fig. 7:**
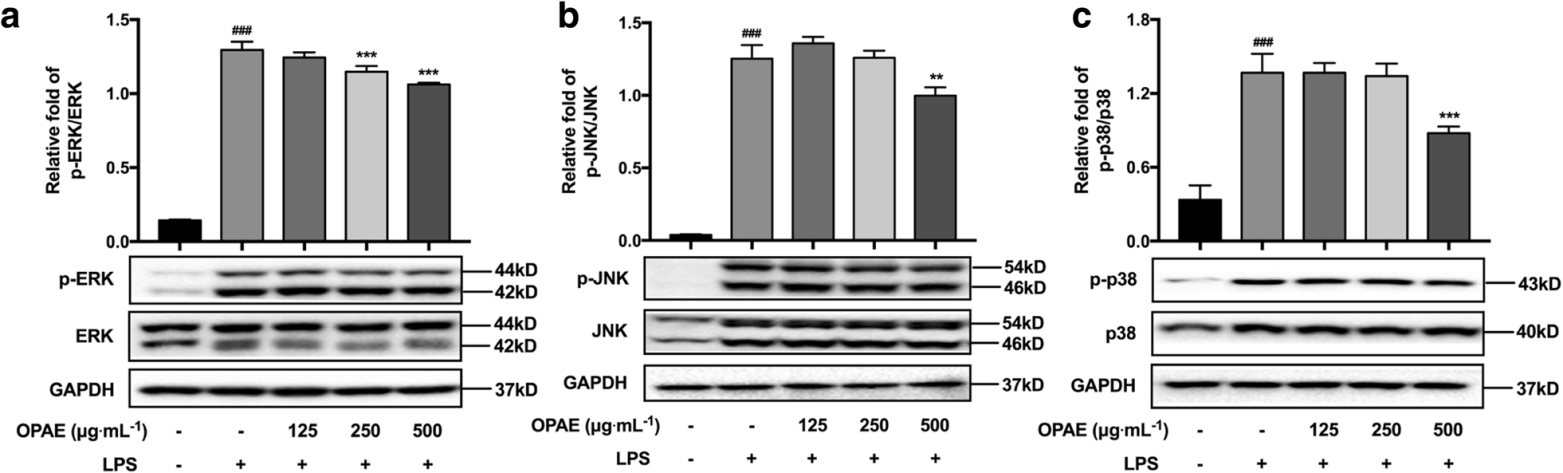
OPAE decreased the phosphorylation of MAPKs in LPS-stimulated RAW 264.7 macrophages. Cells were pre-treated with OPAE at different concentrations (125, 250, and 500 mg mL^−1^) for 4 h and then stimulated with LPS (1 µg mL^−1^) for another 30 min. The protein levels of **a** ERK and p-ERK, **b** JNK and p-JNK, **c** p38 and p-p38 were determined by western blotting (n = 3), GAPDH was used as internal control. Data are expressed as mean ± S.D. ^###^*p *<0.001 vs. Ctrl. group, **p <0.01, ***p <0.001 vs. LPS group

MAPKs signaling pathway is one of key regulators of inflammation, the phosphorylation levels of ERK, p38 and JNK was dramatically increased after LPS activation with expectation (Fig. 7, *p *< 0.001); OPAE concentration-dependently reduced LPS-stimulated phosphorylation of ERK expression, the relative fold decrease of pERK/ERK was 1.24 ± 0.03, 1.15 ± 0.04 (*p *< 0.001) and 1.06 ± 0.01 (*p *< 0.001), respectively. While the phosphorylation of JNK and p38 were also affected by OPAE treatment at 500 μg mL^−1^, the inhibitory rates were 20.36% (*p *< 0.01) and 35.83% (*p *< 0.001), respectively.

## Discussion

Nitric oxide, a gaseous signaling molecule, functions as a vital biological messenger in the pathogenesis of inflammation, which gives antagonistic action under normal physiological conditions but an accelerated regulation in abnormally overabundant states [21]. The biosynthesis of NO is catalyzed by a family of nitric oxide synthase (NOS) enzymes, three different isoforms of which have been characterized as neuronal NOS (nNOS), endothelial NOS (eNOS) and inducible NOS (iNOS) [22]. High concentration of NO generated by exposure to infectious stimulus or immunologically related diseases mediates functional activities of many immune and inflammatory cells [23] including monocytes/macrophages, T lymphocyte, neutrophils, natural killer cells, fibroblasts, osteoclasts, and endothelial cells, through excessive cytokine and matrix metalloproteinase productions, oxidative reactions, mitochondrial dysfunctions, cell apoptosis and death [24–26]. Since Stuehr and Marletta [27] reported that nitrite and nitrate was produced by mouse macrophages in response to bacterial lipopolysaccharide, among which RAW 264.7 cells has been applied as one of the frequently-used models for the screening of natural products for anti-inflammatory agents [28, 29], and abundant herb extractions from CHM showed inhibitory effects on NO secretion. In murine macrophage RAW264.7 cells, LPS activation alone has been successfully proven to the increase of NO level (Fig. 3b), iNOS protein (Fig. 3c) and mRNA expression (Fig. 3d). Taking into account together, the anti-inflammatory therapeutic potential of five extracts isolated from RPA–RAM herb pair using different solvents were evaluated by their NO inhibition rate in RAW 264.7 cells. Extracts 2 (OPAE, 25% ethanol/water extracted system) exhibited the most prominently inhibitory activity with an IC_50_ value of 236.1 μg mL^−1^, and was selected for further mechanism studies. In the present study, our results indicated OPAE concentration-dependently decreased NO production and iNOS protein level, and mRNA expression of iNOS was additionally down-regulated at high concentration. This suggested that the NO/iNOS was a critical target for inflammation suppression of OPAE as well.

Pro-inflammatory cytokines like TNF-α, IL-6 and MCP-1 secreted by activated macrophages have been proven involved in the initiation and regression of immune response with exposure to LPS [30, 31]. In this study, data revealed the decrease of mRNA expression of TNF-α, IL-6 and MCP-1 with OPAE treatment at 500 μg mL^−1^. Interestingly, OPAE strongly reduced TNF-α and MCP-1 production at concentrations of up to 500 μg mL^−1^, but concentration-dependently attenuated IL-6 release, indicating the different dose–effect relationships among OPAE and three inflammatory factors. Since the receptors of IL-6 [32], TNF-α [33] and MCP-1 [34] were different, the affinities between chemical groups of OPAE and the receptors of three inflammatory factors might correspondingly be different. It has been reported that exogenous TNF-α could upregulate SOCS3 [35], which could stop IL-6 signaling in a negative feedback loop [36]. Thus at the concentration of 250 μg mL^−1^, OPAE didn’t reduce the TNF-α, the downregulated level of IL-6 might also be the result of this TNF-α/IL-6 feedback loop. In this term, MCP-1 secretion might be in response to the regulation of TNF-α, which was in consistent with a previous study [37]. This hypothesis could be evidenced by the treatment of OPAE at the concentration of 500 μg mL^−1^, while both the TNF-α and MCP-1 were reduced. Meanwhile, the LPS-activated NF-κB and MAPK pathways could be partially suppressed by OPAE at the highest concentration, indicating that the decrease of TNF-α and MCP-1 might be caused by the downregulation of p65 and MAPK. The effects of OPAE on p-IκBα and IκBα protein levels was found in a dose-dependent way, which might be relevant to that of IL-6. Additionally, the concentration-dependent decrease of IL-6 with OPAE treatment suggested that it might be regulating the related molecules through JAK/STAT pathway, further studies needed to be conducted in the future. Our results indicated that OPAE suppressed LPS-induced inflammation through the down-regulation of the pro-inflammatory cytokines.

The excessive pro-inflammatory mediators are mainly caused by activated transcriptional factors in the development of inflammation progress, among these regulations, NF-κB play an important role in macrophages [38]. External stimuli caused the phosphorylated activation and degradation of IκBα, leading to NF-κB dissociation from the complex, which is composed by homodimer or heterodimer of NF-κB and IκBα, free NF-κB p65 translocated into nuclei for transcriptional regulation of inflammation-associated genes [39, 40]. On the other hand, the phosphorylation and activation of MAPKs have also been reported to promote the phosphorylation of IκBα, NF-κB activation, nuclear transport and subsequent transcriptional regulation [41, 42]. Aggravative immune responses also involves in accumulated pro-inflammatory cytokines promoted by the activation of the MAPK pathway [43]. The results in present study showed that OPAE reduced p65 expression and restricted its nuclear distribution, whereas the expression of p-p65 was not affected. Corresponding to suppressed pro-inflammatory mediators, the significant inhibitory effects on pERK, pJNK and p38, on phosphorylation and degradation of IκBα was obtained in LPS-induced RAW 264.7 cells with OPAE pretreatment, suggesting that OPAE obstructed cascading inflammatory responses via inhibiting MAPKs signaling and NF-κB pathway.

## Conclusion

In the present study, we found that OPAE significantly suppressed the pro-inflammatory mediators such as NO, iNOS, TNF-α, IL-6 and MCP-1 in LPS-stimulated RAW 264.7 macrophages. The anti-inflammatory potentials of OPAE were demonstrated through down-regulating phosphorylated IκBα, decreasing IκBα degradation, preventing NF-κB p65 translocation into nucleus and suppressing MAPKs phosphorylation. Taken together, these results give supporting supplements for the RAM-RPA herb pair in traditional applications, and suggest that OPAE might be useful in inflammation-associated diseases. However, further in vivo experiments and clinical trials are still necessary to confirm the anti-inflammatory activities of OPAE.

## Additional file

**Additional file 1.** Minimum Standards of Reporting Checklist.
